# Supplementary material for: The Evolution and Characterization of the RNA Interference Pathways in Lophotrochozoa
Source: Genome Biol Evol. 2024 May 7;16(5):evae098. doi: 10.1093/gbe/evae098 (PMC11114477; doi:10.1093/gbe/evae098)
Supplement: evae098_Supplementary_Data [file evae098_supplementary_data.zip › Supplementary_materials.zip!/selected_miRNAs/NoGe_selected_miRNAs/mirdeep_pdf_files/NMRB01001254.1_Notospermus_geniculatus_strain_Ushimado_scaffold1254,_whole_genome_shotgun_sequence_26696.pdf]

Provisional ID	:	NMRB01001254.1_Notospermus_geniculatus_strain_Ushimado_scaffold1254,_whole_genome_shotgun_sequence_26696
Score total	:	62177.2
Score for star read(s)	:	3.9
Score for read counts	:	62169.2
Score for mfe	:	1.1
Score for randfold	:	0
Score for cons. seed	:	3
Total read count	:	121954
Mature read count	:	121926
Loop read count	:	0
Star read count	:	28

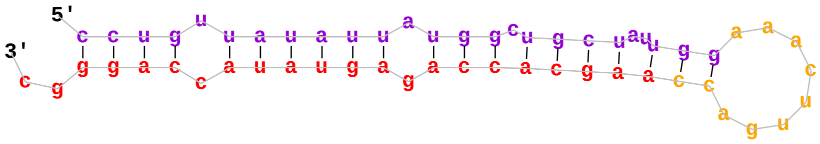

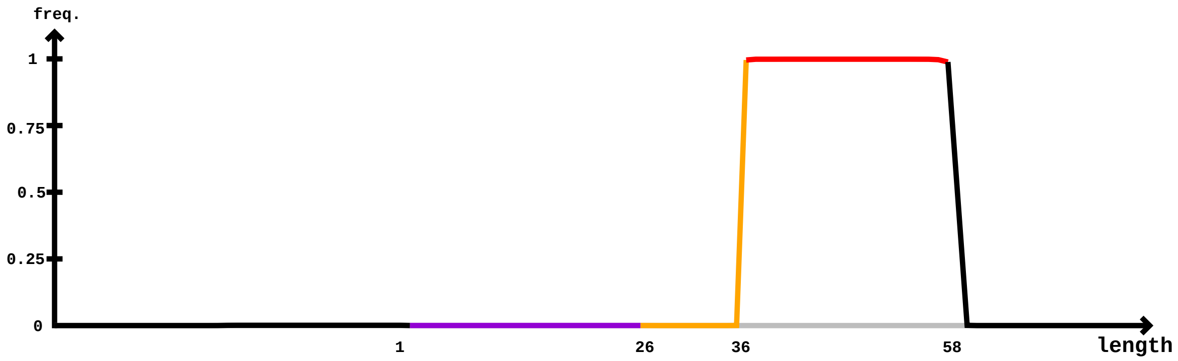

Star Mature

```
5'- auaccuggaaucaagaugugcauccuaucuaguguccuguuauauuauggcugcuauuggaaacuugaccaagcaccagaguauaccagggcagcacugggacacccacagc  -3'   obs
    auaccuggaaucaagaugugcauccuaucuaguguccuguuauauuauggcugcuauuggaaacuugaccaagcaccagaguauaccagggcagcacugggacacccacagc         exp
    ................((((..(((((.....(((((((.((((((.(((.((((..(((........)))))))))).)))))).)))))))....)))))....))))..   reads   mm   sample
    ................ugugcauccuaucuagAgu.............................................................................   1       1    Mm1
    ................ugugcauccuaucuagugu.............................................................................   13      0    Mm1
    ...................................ccuguuauauuauggcugcuauugg....................................................   9       0    Mm1
    ......................................................................aagcaccagaguauaccagg......................   33      0    Mm1
    ......................................................................aagcaccagaAuauaccagg......................   1       1    Mm1
    ......................................................................aagcaccagaguauaccUggg.....................   1       1    Mm1
    ......................................................................aagcaccagaguauCccaggg.....................   1       1    Mm1
    ......................................................................aagcaccagaguauacUaggg.....................   1       1    Mm1
    ......................................................................aagcaccagaguauaccaggg.....................   252     0    Mm1
    ......................................................................aagcaccagaguauacGaggg.....................   3       1    Mm1
    ......................................................................aagcaccagaguauaccGggg.....................   2       1    Mm1
    ......................................................................aagcaccagaguauaccCggg.....................   1       1    Mm1
    ......................................................................aaCcaccagaguauaccagggc....................   1       1    Mm1
    ......................................................................aagcaccagaguauaccaUggc....................   1       1    Mm1
    ......................................................................aagcaccagaguauacUagggc....................   14      1    Mm1
    ......................................................................aagcaccagaUuauaccagggc....................   5       1    Mm1
    ......................................................................aagcaccagGguauaccagggc....................   4       1    Mm1
    ......................................................................aagcaccagaguauaccUgggc....................   10      1    Mm1
    ......................................................................aGgcaccagaguauaccagggc....................   1       1    Mm1
    ......................................................................aagcacAagaguauaccagggc....................   2       1    Mm1
    ......................................................................aagcaccagaCuauaccagggc....................   1       1    Mm1
    ......................................................................aagcaccaUaguauaccagggc....................   5       1    Mm1
    ......................................................................aagcaccagagCauaccagggc....................   1       1    Mm1
    ......................................................................aagcaccagaAuauaccagggc....................   7       1    Mm1
    ......................................................................Nagcaccagaguauaccagggc....................   2       1    Mm1
    ......................................................................aagcaccagaguauaccCgggc....................   7       1    Mm1
    ......................................................................aagcaccaAaguauaccagggc....................   1       1    Mm1
    ......................................................................aagcaccagaguauaccagAgc....................   1       1    Mm1
    ......................................................................aagcaccagaguauaccagggA....................   8       1    Mm1
    ......................................................................Uagcaccagaguauaccagggc....................   4       1    Mm1
    ......................................................................aagcaccagaguauaccagggc....................   3118    0    Mm1
    ......................................................................aagcaccagaguauacAagggc....................   10      1    Mm1
    ......................................................................aagcGccagaguauaccagggc....................   1       1    Mm1
```

```
                                   Star                                Mature
auaccuggaaucaagaugugcauccuaucuagugucuguuauauuauggcugcuauuggaaacuugaccaagcaccagaguauaccagggcagcacugggacacccacagc
......................................................................aagcacUagaguauaccagggc....................   1      1      Mm1
......................................................................aaUcaccagaguauaccagggc....................   2      1      Mm1
......................................................................aagAaccagaguauaccagggc....................   1      1      Mm1
......................................................................aagUaccagaguauaccagggc....................   1      1      Mm1
......................................................................aagcaccagaguauaccGgggc....................   12     1      Mm1
......................................................................aagcaccagaguauaccagggU....................   25     1      Mm1
......................................................................aagcaccagaguauacGagggc....................   7      1      Mm1
......................................................................aagcaccGgaguauaccagggc....................   1      1      Mm1
......................................................................aagcaccagaguauaccagggcU...................   18     1      Mm1
......................................................................aagcaccagaguauaccagggAa...................   8      1      Mm1
......................................................................aagcaccagaguauacUagggca...................   1      1      Mm1
......................................................................aagcaccagaguauaccagggca...................   74     0      Mm1
......................................................................aagcaccagaguauaccagggcaA..................   2      1      Mm1

................ugugcauccuaucuagug..............................................................................   1      0      Mm2
................ugugcauccuaucuagugG.............................................................................   3      1      Mm2
................ugugcauccuaucuagugu.............................................................................   7      0      Mm2
.................gugcauccuaucuagugu.............................................................................   1      0      Mm2
...................................ccuguuauauuauggcugcuauugg....................................................   7      0      Mm2
...................................ccuguuauauuauggcugcuauugga...................................................   3      0      Mm2
....................................cuguuauauuauggcugcuauugga...................................................   1      0      Mm2
..................................................................gaUcaagcaccagaguauaccagggc....................   1      1      Mm2
.....................................................................caagcaccagaguauaccagg......................   1      0      Mm2
.....................................................................caagcaccagaguauaccagggc....................   26     0      Mm2
.....................................................................caagcacAagaguauaccagggc....................   1      1      Mm2
......................................................................aagcaccagaguauaccag.......................   4      0      Mm2
......................................................................aagcaccagaguauaccagg......................   48     0      Mm2
......................................................................Uagcaccagaguauaccaggg.....................   1      1      Mm2
......................................................................aagcaccagaguauaccaUgg.....................   1      1      Mm2
......................................................................aagcaccagaguauaccCggg.....................   2      1      Mm2
......................................................................aagcaccagUguauaccaggg.....................   1      1      Mm2
......................................................................aagcaccagaguauacUaggg.....................   1      1      Mm2
......................................................................aagcaccagaguauacAaggg.....................   2      1      Mm2
......................................................................aagcaccagaguauaccaggC.....................   6      1      Mm2
......................................................................aagcaccagaguauaccaggg.....................   246    0      Mm2
......................................................................aagcaccagaguauaccNgggc....................   1      1      Mm2
......................................................................aagcaccagaguauaccaggUc....................   18     1      Mm2
......................................................................aagcaccagagCauaccagggc....................   7      1      Mm2
......................................................................aaAcaccagaguauaccagggc....................   25     1      Mm2
......................................................................aagcacAagaguauaccagggc....................   1      1      Mm2
......................................................................Cagcaccagaguauaccagggc....................   1      1      Mm2
......................................................................aagcaccagaguGuaccagggc....................   6      1      Mm2
......................................................................aagcaccagaguauaccUgggc....................   222    1      Mm2
......................................................................aagcaccagaCuauaccagggc....................   79     1      Mm2
......................................................................aagcaccagaguauaccaggCc....................   3      1      Mm2
......................................................................aagcaccagaguaAaccagggc....................   13     1      Mm2
......................................................................aagcGccagaguauaccagggc....................   10     1      Mm2
......................................................................aagcaccagaguauaccagggc....................   73220  0      Mm2
......................................................................aagcaccagCguauaccagggc....................   4      1      Mm2
......................................................................aagcacGagaguauaccagggc....................   6      1      Mm2
......................................................................aagcaccagaguCuaccagggc....................   1      1      Mm2
......................................................................aagcaccagaguauaGcagggc....................   1      1      Mm2
......................................................................aagcaccagagGauaccagggc....................   4      1      Mm2
......................................................................aagcaAcagaguauaccagggc....................   4      1      Mm2
......................................................................aagcaccagaguauaccagUgc....................   14     1      Mm2
......................................................................aagcaccagaAuauaccagggc....................   111    1      Mm2
......................................................................aaNcaccagaguauaccagggc....................   1      1      Mm2
......................................................................aagcaccagagAauaccagggc....................   8      1      Mm2
......................................................................aagcaccCgaguauaccagggc....................   1      1      Mm2
......................................................................aagcaccagaguauaccGgggc....................   219    1      Mm2
......................................................................aagcaccagaguaGaccagggc....................   4      1      Mm2
......................................................................aagGaccagaguauaccagggc....................   2      1      Mm2
......................................................................aagcCccagaguauaccagggc....................   7      1      Mm2
......................................................................aagcacNagaguauaccagggc....................   8      1      Mm2
......................................................................aagcaccagaguUuaccagggc....................   3      1      Mm2
......................................................................aagcacUagaguauaccagggc....................   19     1      Mm2
......................................................................aagcaGcagaguauaccagggc....................   3      1      Mm2
......................................................................Gagcaccagaguauaccagggc....................   9      1      Mm2
......................................................................aagcaccagaguauacGagggc....................   105    1      Mm2
......................................................................aagcaccaUaguauaccagggc....................   43     1      Mm2
```

Star **Mature**

```
auaccuggaaucaagaugugcauccuaucuaguguccuguuauauuauggcugcuauuggaaacuugaccaagcaccagaguauaccagggcagcacugggacacccacagc
......................................................................aagAaccagaguauaccagggc....................   12    1   Mm2
......................................................................aagcaccagaguauaccCgggc....................   206   1   Mm2
......................................................................aagcaccaAaguauaccagggc....................   9     1   Mm2
......................................................................aagcaccagaUuauaccagggc....................   59    1   Mm2
......................................................................aagcaccagUguauaccagggc....................   15    1   Mm2
......................................................................aCgcaccagaguauaccagggc....................   3     1   Mm2
......................................................................aagcaccagaguauaccagggU....................   103   1   Mm2
......................................................................aagcaccagaguauaccagCgc....................   3     1   Mm2
......................................................................aaCcaccagaguauaccagggc....................   8     1   Mm2
......................................................................aagcaccagGguauaccagggc....................   15    1   Mm2
......................................................................aagcaccagaguauaccaAggc....................   7     1   Mm2
......................................................................aagUaccagaguauaccagggc....................   8     1   Mm2
......................................................................aagcaccagaguauaccagggA....................   13    1   Mm2
......................................................................aagcaccagaguauacUagggc....................   237   1   Mm2
......................................................................aagcaccagaguauaccagAgc....................   7     1   Mm2
......................................................................aagcaccagaguauacAagggc....................   327   1   Mm2
......................................................................aagcaccagaguauaAcagggc....................   3     1   Mm2
......................................................................aagcaccagaguauUccagggc....................   8     1   Mm2
......................................................................aagcaccagaguauGccagggc....................   3     1   Mm2
......................................................................aagcaccUgaguauaccagggc....................   5     1   Mm2
......................................................................Nagcaccagaguauaccagggc....................   28    1   Mm2
......................................................................aagcaccagaguauaccagggG....................   14    1   Mm2
......................................................................aGgcaccagaguauaccagggc....................   7     1   Mm2
......................................................................aagcaccagaguaCaccagggc....................   10    1   Mm2
......................................................................aUgcaccagaguauaccagggc....................   4     1   Mm2
......................................................................aagcaccagaguauaccaggAc....................   12    1   Mm2
......................................................................Uagcaccagaguauaccagggc....................   45    1   Mm2
......................................................................aaUcaccagaguauaccagggc....................   25    1   Mm2
......................................................................aagcaccagaguauaccaUggc....................   8     1   Mm2
......................................................................aagcaNcagaguauaccagggc....................   9     1   Mm2
......................................................................aagcaccGgaguauaccagggc....................   13    1   Mm2
......................................................................aagcaccagaguauaUcagggc....................   16    1   Mm2
......................................................................aagcaccagaguauaccaCggc....................   5     1   Mm2
......................................................................aagcaUcagaguauaccagggc....................   10    1   Mm2
......................................................................aagcUccagaguauaccagggc....................   12    1   Mm2
......................................................................aagcaccagaguauaccagCgca...................   1     1   Mm2
......................................................................aagcaccagaguauaccagggcU...................   330   1   Mm2
......................................................................aagcaccagaguauaccaggUca...................   1     1   Mm2
......................................................................aagcaccagaguauacAagggca...................   2     1   Mm2
......................................................................aagcaccagaguauaccagggcG...................   9     1   Mm2
......................................................................aagcaccagaguauaccagggca...................   124   0   Mm2
......................................................................aagcaccagaCuauaccagggca...................   1     1   Mm2
......................................................................aagcaccagaguauaccGgggca...................   3     1   Mm2
......................................................................aagcaccagaguauaccagggcC...................   8     1   Mm2
......................................................................aagcaccagaguauaccUgggca...................   1     1   Mm2
......................................................................aagcaccagaguauaccagUgca...................   1     1   Mm2
......................................................................aagcaccagaAuauaccagggca...................   1     1   Mm2
......................................................................aagcaccagaguauaccagggcaU..................   20    1   Mm2
......................................................................aagcaccagaguauaccagggcUg..................   6     1   Mm2
......................................................................aagcaccagaguauaccagggcaA..................   6     1   Mm2
......................................................................aagcaccagaguauaccagggcCg..................   1     1   Mm2
.......................................................................agcaccagaguauaccCgggc....................   3     1   Mm2
.......................................................................agcaccagaguauaAcagggc....................   1     1   Mm2
.......................................................................agcaccagCguauaccagggc....................   2     1   Mm2
.......................................................................agcaccagaguauacUagggc....................   1     1   Mm2
.......................................................................agcaccaAaguauaccagggc....................   1     1   Mm2
.......................................................................agcaccagaguauaccaCggc....................   1     1   Mm2
.......................................................................agcaccagagAauaccagggc....................   2     1   Mm2
.......................................................................agcaccagaguauaccagggc....................   217   0   Mm2
.......................................................................agcaccagaguauaccaAggc....................   1     1   Mm2
.......................................................................agcaccagaguauaccagggca...................   1     0   Mm2
........................................................................gcaccagaguauaccagggc....................   1     0   Mm2
..........................................................................accagaguauaccagggc....................   3     0   Mm2

................ugugcauccuaucuagugG.............................................................................   2     1   Fm1
................ugugcauccuaucuagugu.............................................................................   12    0   Fm1
................ugGgcauccuaucuagugu.............................................................................   1     1   Fm1
................ugugcauccuaucuagCgu.............................................................................   1     1   Fm1
.................gugcauccuaucuagugu.............................................................................   5     0   Fm1
...................................ccuguuauauuauggcug...........................................................   2     0   Fm1
```

```
                                   Star                             Mature
auaccuggaaucaagaugugcauccuaucuaguguccuguuauauuauggcugcuauuggaaacuugaccaagcaccagaguauaccagggcagcacugggacacccacagc
...................................ccuguuauauuauggcugcuauugg....................................................	2	0	Fm1
.....................................................................caagcaccagaguauaccagggc....................	1	0	Fm1
......................................................................aagcaccagaguauaccagg......................	15	0	Fm1
......................................................................aagcaccagaguauacAaggg.....................	1	1	Fm1
......................................................................aagcaccagaguauaccaggU.....................	2	1	Fm1
......................................................................aagcaccagaguauaccaggg.....................	126	0	Fm1
......................................................................aagcaccagaguauaccGggg.....................	1	1	Fm1
......................................................................aagcaccagaguauaccUggg.....................	1	1	Fm1
......................................................................aagcaNcagaguauaccagggc....................	2	1	Fm1
......................................................................aagcaccagaguauUccagggc....................	1	1	Fm1
......................................................................aGgcaccagaguauaccagggc....................	5	1	Fm1
......................................................................aagcaccagaguauaccagUgc....................	2	1	Fm1
......................................................................aagcUccagaguauaccagggc....................	3	1	Fm1
......................................................................aaAcaccagaguauaccagggc....................	5	1	Fm1
......................................................................aUgcaccagaguauaccagggc....................	1	1	Fm1
......................................................................aagcaccagUguauaccagggc....................	6	1	Fm1
......................................................................aagcaccagaguauaccagggc....................	15552	0	Fm1
......................................................................aagcaccagaguauaccCgggc....................	46	1	Fm1
......................................................................aagcaccagCguauaccagggc....................	2	1	Fm1
......................................................................aagcCccagaguauaccagggc....................	1	1	Fm1
......................................................................aagcaccagagAauaccagggc....................	2	1	Fm1
......................................................................Cagcaccagaguauaccagggc....................	1	1	Fm1
......................................................................aagGaccagaguauaccagggc....................	1	1	Fm1
......................................................................aagcacUagaguauaccagggc....................	2	1	Fm1
......................................................................aagcaccagaguaCaccagggc....................	1	1	Fm1
......................................................................aagcaccGgaguauaccagggc....................	5	1	Fm1
......................................................................aagcaccagaCuauaccagggc....................	11	1	Fm1
......................................................................aagcaccagaguauaccagggG....................	3	1	Fm1
......................................................................aagAaccagaguauaccagggc....................	4	1	Fm1
......................................................................aagcaccagaguaGaccagggc....................	2	1	Fm1
......................................................................aaUcaccagaguauaccagggc....................	2	1	Fm1
......................................................................aagcaccagaguauacAagggc....................	67	1	Fm1
......................................................................aagcaccagaguauaccagggU....................	28	1	Fm1
......................................................................aagcacNagaguauaccagggc....................	1	1	Fm1
......................................................................aagcaccagaguauaccGgggc....................	59	1	Fm1
......................................................................Nagcaccagaguauaccagggc....................	11	1	Fm1
......................................................................aagcGccagaguauaccagggc....................	1	1	Fm1
......................................................................aagcaccagaguauaccagCgc....................	1	1	Fm1
......................................................................aagcaccagGguauaccagggc....................	5	1	Fm1
......................................................................aagcacAagaguauaccagggc....................	2	1	Fm1
......................................................................aagcaccagaguauaccaggCc....................	2	1	Fm1
......................................................................aCgcaccagaguauaccagggc....................	3	1	Fm1
......................................................................aagcaccagaguauacGagggc....................	11	1	Fm1
......................................................................aagcaccagaguaAaccagggc....................	1	1	Fm1
......................................................................Gagcaccagaguauaccagggc....................	4	1	Fm1
......................................................................aagcaccagaAuauaccagggc....................	30	1	Fm1
......................................................................aagcaccagaguauaccaggUc....................	6	1	Fm1
......................................................................aagUaccagaguauaccagggc....................	4	1	Fm1
......................................................................aagcaccaUaguauaccagggc....................	11	1	Fm1
......................................................................aagcaccagaguGuaccagggc....................	2	1	Fm1
......................................................................aagcaccagaguauaccUgggc....................	23	1	Fm1
......................................................................aaCcaccagaguauaccagggc....................	3	1	Fm1
......................................................................aagcaccagaguauaccagggA....................	4	1	Fm1
......................................................................aagcaccagaguauaccagAgc....................	2	1	Fm1
......................................................................aagcaccagagCauaccagggc....................	1	1	Fm1
......................................................................Uagcaccagaguauaccagggc....................	10	1	Fm1
......................................................................aagcaccagaUuauaccagggc....................	7	1	Fm1
......................................................................aagcaccCgaguauaccagggc....................	1	1	Fm1
......................................................................aagcaccagaguauaccaUggc....................	5	1	Fm1
......................................................................aagcaccagaguauacUagggc....................	38	1	Fm1
......................................................................aagcaccagaguauaUcagggc....................	12	1	Fm1
......................................................................aagcaccagaguauaccagggAa...................	4	1	Fm1
......................................................................aagcaccagaguauaccagggcG...................	16	1	Fm1
......................................................................aagcaccagaguauaccUgggca...................	1	1	Fm1
......................................................................aagcaccagaguauaccagggcU...................	237	1	Fm1
......................................................................aagcaccagaguauaccCgggca...................	1	1	Fm1
......................................................................aagcaccagaguauaccagggca...................	93	0	Fm1
......................................................................aagcaccagaguauaccagggcC...................	1	1	Fm1
......................................................................aagcaccagaguauaccagggcaU..................	29	1	Fm1
......................................................................aagcaccagaguauaccagggcaA..................	1	1	Fm1
```

```
                                   Star                            Mature
auaccuggaaucaagaugugcauccuaucuaguguccuguuauauuauggcugcuauuggaaacuugaccaagcaccagaguauaccagggcagcacugggacacccacagc
.......................................................................agcaccagagCauaccagggc....................    1       1       Fm1
.......................................................................agcaccagagAauaccagggc....................    1       1       Fm1
.......................................................................agcaccagaguauaccagggc....................    18      0       Fm1

.....................................................................caagcaccagaguauaccagggc....................    4       0       Fm2
.....................................................................caagcaccagaguauaccCgggc....................    1       1       Fm2
......................................................................aagcaccagaguauaccagg......................    4       0       Fm2
......................................................................aagcaccagaguauaccUggg.....................    1       1       Fm2
......................................................................aagcaccagaguauaccaggU.....................    6       1       Fm2
......................................................................aagcaccagaguauacUaggg.....................    1       1       Fm2
......................................................................aagcaccagaguauaccaggg.....................    32      0       Fm2
......................................................................aagcaccagaguauaccagUg.....................    1       1       Fm2
......................................................................aagcaccagaguauacUagggc....................    20      1       Fm2
......................................................................aagcaccaAaguauaccagggc....................    1       1       Fm2
......................................................................aagcaccagaguauaGcagggc....................    1       1       Fm2
......................................................................Uagcaccagaguauaccagggc....................    2       1       Fm2
......................................................................aagcGccagaguauaccagggc....................    1       1       Fm2
......................................................................aagcCccagaguauaccagggc....................    1       1       Fm2
......................................................................aagcaccagaguauacGagggc....................    9       1       Fm2
......................................................................aagcaccagaguauaccagUgc....................    3       1       Fm2
......................................................................aagcUccagaguauaccagggc....................    1       1       Fm2
......................................................................aagcaccagaguauaUcagggc....................    1       1       Fm2
......................................................................aagcaccagGguauaccagggc....................    2       1       Fm2
......................................................................aagcaccagaUuauaccagggc....................    6       1       Fm2
......................................................................aagcaccagaguauacNagggc....................    1       1       Fm2
......................................................................aagcaccagaguauaccaUggc....................    1       1       Fm2
......................................................................aagcacUagaguauaccagggc....................    1       1       Fm2
......................................................................aagcaccagaCuauaccagggc....................    5       1       Fm2
......................................................................aagcaccagaguauaAcagggc....................    1       1       Fm2
......................................................................aagcaccagaguauaccaggAc....................    2       1       Fm2
......................................................................aagcaccagaguauaccagggc....................    6204    0       Fm2
......................................................................aaUcaccagaguauaccagggc....................    1       1       Fm2
......................................................................aagcaccagaguauaccagggA....................    6       1       Fm2
......................................................................aagcaccagaAuauaccagggc....................    12      1       Fm2
......................................................................aUgcaccagaguauaccagggc....................    3       1       Fm2
......................................................................aagcacAagaguauaccagggc....................    1       1       Fm2
......................................................................aagcaccagaguauacAagggc....................    28      1       Fm2
......................................................................Gagcaccagaguauaccagggc....................    3       1       Fm2
......................................................................aagcaccagaguaCaccagggc....................    8       1       Fm2
......................................................................Nagcaccagaguauaccagggc....................    1       1       Fm2
......................................................................aagcaccagaguauaccUgggc....................    22      1       Fm2
......................................................................aagcaccagaguauaccGgggc....................    24      1       Fm2
......................................................................aagcaccagaguauaccagAgc....................    1       1       Fm2
......................................................................aagcaUcagaguauaccagggc....................    2       1       Fm2
......................................................................aagcaccagaguauaccagggG....................    2       1       Fm2
......................................................................aagcaccGgaguauaccagggc....................    1       1       Fm2
......................................................................aagcaccaUaguauaccagggc....................    14      1       Fm2
......................................................................aagcaccagaguauaccCgggc....................    17      1       Fm2
......................................................................aagcaccUgaguauaccagggc....................    2       1       Fm2
......................................................................aagcacGagaguauaccagggc....................    1       1       Fm2
......................................................................aagcaNcagaguauaccagggc....................    2       1       Fm2
......................................................................aagcaAcagaguauaccagggc....................    1       1       Fm2
......................................................................aagcaccagaguauaccaAggc....................    1       1       Fm2
......................................................................aagcaccagaguauaccagggU....................    14      1       Fm2
......................................................................aagcaccagaguauaccaCggc....................    1       1       Fm2
......................................................................aaAcaccagaguauaccagggc....................    2       1       Fm2
......................................................................aagcaccagaguauaccagCgc....................    1       1       Fm2
......................................................................aagcaccagaguauaccagggca...................    61      0       Fm2
......................................................................aagcaccagaguauaccagggcU...................    24      1       Fm2
......................................................................aagcaccagaguauacUagggca...................    1       1       Fm2
......................................................................aagcaccagaguauaccagggcaU..................    11      1       Fm2
.......................................................................agcaccagagCauaccagggc....................    1       1       Fm2
.......................................................................agcaccagaguauaccagggc....................    15      0       Fm2

................ugugcauccuaucuagugu.............................................................................    4       0       Mm3
.................gugcauccuaucuagugu.............................................................................    2       0       Mm3
...................................ccuguuauauuauggcugcuauugg....................................................    2       0       Mm3
...................................ccuguuauauuauggcugcuauugga...................................................    1       0       Mm3
....................................cuguuauauuauggcugcuauugg....................................................    1       0       Mm3
.....................................................................caagcaccagaguauaccaggU.....................    1       1       Mm3
```

Star Mature

auaccuggaaucaagaugugcauccuaucuagugu**ccuguuauauuauggcugcuauugg**aaacuugacc**aagcaccagaguauaccagggc**agcacugggacacccacagc

....caagcaccagaguauaccaggg.....................	1	0	Mm3
....caagcaccagaguauaccagggc....................	2	0	Mm3
....caagcaccagaguauaccagggcag..................	1	0	Mm3
....aagcaccagaguauaccag.......................	1	0	Mm3
....aagcaccagaguauaccagg......................	18	0	Mm3
....aagcaccagaguauaccaggU.....................	3	1	Mm3
....aagcaccagaCuauaccaggg.....................	1	1	Mm3
....aagAaccagaguauaccaggg.....................	1	1	Mm3
....aagcaccagaguauaccaggC.....................	1	1	Mm3
....aagcaccagaguauaccaggg.....................	128	0	Mm3
....aagcaccagaguauaccaggA.....................	1	1	Mm3
....aagcaccagaguauaccagggU....................	17	1	Mm3
....aagcaccagaguauaUcagggc....................	1	1	Mm3
....aagcaccagaguauaccaUggc....................	1	1	Mm3
....aagcaccagaguauacAagggc....................	27	1	Mm3
....aagcaccagaCuauaccagggc....................	6	1	Mm3
....aCgcaccagaguauaccagggc....................	1	1	Mm3
....aaAcaccagaguauaccagggc....................	1	1	Mm3
....aagcaccagaguauaccUgggc....................	24	1	Mm3
....aagcaccagaUuauaccagggc....................	7	1	Mm3
....aagcaccagaguauaccaggAc....................	1	1	Mm3
....aagcaccagaguauacUagggc....................	21	1	Mm3
....aagcaccagaguauaccGgggc....................	26	1	Mm3
....aagcaccagaguGuaccagggc....................	1	1	Mm3
....aagcaccagaguauaccaggCc....................	2	1	Mm3
....Gagcaccagaguauaccagggc....................	1	1	Mm3
....aagcaccagaguauaccagUgc....................	2	1	Mm3
....aagcaccagaAuauaccagggc....................	16	1	Mm3
....aagcaccagUguauaccagggc....................	2	1	Mm3
....aagcacGagaguauaccagggc....................	1	1	Mm3
....aagcaccagaguauaccagggA....................	2	1	Mm3
....aagcacUagaguauaccagggc....................	2	1	Mm3
....aUgcaccagaguauaccagggc....................	3	1	Mm3
....aagcacNagaguauaccagggc....................	3	1	Mm3
....aagcaccagaguaAaccagggc....................	1	1	Mm3
....aagcaccagCguauaccagggc....................	1	1	Mm3
....aagcaccagaguauaccCgggc....................	26	1	Mm3
....aagcaccCgaguauaccagggc....................	1	1	Mm3
....aaCcaccagaguauaccagggc....................	1	1	Mm3
....Nagcaccagaguauaccagggc....................	5	1	Mm3
....aagcaUcagaguauaccagggc....................	2	1	Mm3
....aagcaccagaguaCaccagggc....................	1	1	Mm3
....aagcaccagaguauaccagggG....................	1	1	Mm3
....aagcaccagaguauaccagAgc....................	2	1	Mm3
....aagcaccagaguauaccagggc....................	8173	0	Mm3
....aagcaccagaguauaGcagggc....................	1	1	Mm3
....aagcaccagaguauUccagggc....................	2	1	Mm3
....aagcaccaUaguauaccagggc....................	16	1	Mm3
....Uagcaccagaguauaccagggc....................	8	1	Mm3
....aagcaccUgaguauaccagggc....................	2	1	Mm3
....aagcaccagaguauaccaggUc....................	5	1	Mm3
....aagcaccagaguauGccagggc....................	1	1	Mm3
....Cagcaccagaguauaccagggc....................	1	1	Mm3
....aagcaccagaguauacGagggc....................	15	1	Mm3
....aagcaccagaguaGaccagggc....................	3	1	Mm3
....aaUcaccagaguauaccagggc....................	3	1	Mm3
....aGgcaccagaguauaccagggc....................	1	1	Mm3
....aagcaccagaguauaccagggcU...................	96	1	Mm3
....aagcaccagaguauaccagggAa...................	1	1	Mm3
....aagcaccagaguauaccagggcC...................	1	1	Mm3
....aagcaccagaguauaccagCgca...................	1	1	Mm3
....aagcaccagaguauaccagggca...................	25	0	Mm3
....aagcaccagaguauaccagggcG...................	2	1	Mm3
....aagcaccagaguauaccagggcaU..................	4	1	Mm3
....aagcaccaCaguauaccagggcag..................	1	1	Mm3
....aagcaccagaguauaccagggcag..................	1	0	Mm3
....aagcaccagaguauaccagggcUg..................	5	1	Mm3
....aagcaccagaguauaccagggcaA..................	3	1	Mm3
....aagcaccagaguauaccagggcagU.................	1	1	Mm3
....agcaccagagCauaccagggc....................	1	1	Mm3

```
                                   Star                                Mature
auaccuggaaucaagaugugcauccuaucuaguguccuguuauauuauggcugcuauuggaaacuugaccaagcaccagaguauaccagggcagcacugggacacccacagc
.......................................................................agcaccagaguauaccagggc....................  23    0  Mm3
........................................................................gcaccagaguauaccaggg.....................  1     0  Mm3
........................................................................gcaccagaguauaccagggc....................  3     0  Mm3
.........................................................................caccagaguauaccagggca...................  1     0  Mm3
..........................................................................accagaguauaccagggc....................  2     0  Mm3

.............agaugugcauccuaucuagugu.............................................................................  1     0  Fm3
...............augugcauccuaucuagug..............................................................................  2     0  Fm3
...............augugcauccuaucuagCg..............................................................................  1     1  Fm3
...............augugcauccuaucuagugu.............................................................................  7     0  Fm3
................ugugcauccuaucuagAgu.............................................................................  1     1  Fm3
................ugugcauccuaucuaguCu.............................................................................  1     1  Fm3
................ugugcauccuaucuagugu.............................................................................  40    0  Fm3
.................gugcauccuaucuagugu.............................................................................  16    0  Fm3
......................................................................aagcaccagaguauacca........................  3     0  Fm3
......................................................................aagcaccagaguauaccag.......................  13    0  Fm3
......................................................................aagcaccagaguauaccUg.......................  1     1  Fm3
......................................................................Uagcaccagaguauaccagg......................  1     1  Fm3
......................................................................aagcaccagaguauaccagg......................  75    0  Fm3
......................................................................aagcaccagaAuauaccagg......................  1     1  Fm3
......................................................................aagcaccagaguauacUaggg.....................  1     1  Fm3
......................................................................aagcaccagaguauaccUggg.....................  1     1  Fm3
......................................................................aagcaccagaguauacAaggg.....................  1     1  Fm3
......................................................................aagcaccagaguauaccaggg.....................  225   0  Fm3
......................................................................aagcaccagaAuauaccaggg.....................  1     1  Fm3
......................................................................aagcaccagaguauaAcaggg.....................  1     1  Fm3
......................................................................aagcacAagaguauaccagggc....................  1     1  Fm3
......................................................................aagcaccUgaguauaccagggc....................  1     1  Fm3
......................................................................aagcaccagaguauaccaggAc....................  3     1  Fm3
......................................................................aagcaccagaCuauaccagggc....................  7     1  Fm3
......................................................................aagcaccagaguauaccagggc....................  8949  0  Fm3
......................................................................aagcaccagaguauaAcagggc....................  4     1  Fm3
......................................................................aagcaccagaguauacUagggc....................  34    1  Fm3
......................................................................aagcaccagUguauaccagggc....................  3     1  Fm3
......................................................................aagcaccagaguauaccagUgc....................  4     1  Fm3
......................................................................aagcaccagaguaGaccagggc....................  1     1  Fm3
......................................................................Gagcaccagaguauaccagggc....................  2     1  Fm3
......................................................................aagcaccagaguaAaccagggc....................  1     1  Fm3
......................................................................aaUcaccagaguauaccagggc....................  2     1  Fm3
......................................................................aagcaccagaguauaccCgggc....................  25    1  Fm3
......................................................................aagcaccagaguauaccGgggc....................  33    1  Fm3
......................................................................Cagcaccagaguauaccagggc....................  1     1  Fm3
......................................................................aagcacGagaguauaccagggc....................  2     1  Fm3
......................................................................aagcaccagagAauaccagggc....................  1     1  Fm3
......................................................................Nagcaccagaguauaccagggc....................  4     1  Fm3
......................................................................aagcaccagaguauaccaggCc....................  1     1  Fm3
......................................................................aagcaccagaguauaccagggA....................  14    1  Fm3
......................................................................aagcaccagaguUuaccagggc....................  1     1  Fm3
......................................................................aagcaccaUaguauaccagggc....................  33    1  Fm3
......................................................................aagcaUcagaguauaccagggc....................  2     1  Fm3
......................................................................aagcaccagaguauaccUgggc....................  31    1  Fm3
......................................................................aagcaccaAaguauaccagggc....................  3     1  Fm3
......................................................................aagcaccagagCauaccagggc....................  5     1  Fm3
......................................................................aagcaccagaguauaccaggUc....................  2     1  Fm3
......................................................................aagcaccagaguauaccaUggc....................  4     1  Fm3
......................................................................aagcaccagaguauaccagggU....................  22    1  Fm3
......................................................................aagcaccagGguauaccagggc....................  2     1  Fm3
......................................................................aagcaccagaguauaccaAggc....................  2     1  Fm3
......................................................................aagcUccagaguauaccagggc....................  2     1  Fm3
......................................................................aagcaccagaUuauaccagggc....................  8     1  Fm3
......................................................................aagcaccagaAuauaccagggc....................  12    1  Fm3
......................................................................aagcaccGgaguauaccagggc....................  2     1  Fm3
......................................................................aagcaccagaguGuaccagggc....................  8     1  Fm3
......................................................................aagcaccagaguauaUcagggc....................  8     1  Fm3
......................................................................aaAcaccagaguauaccagggc....................  5     1  Fm3
......................................................................aagcacNagaguauaccagggc....................  2     1  Fm3
......................................................................Uagcaccagaguauaccagggc....................  7     1  Fm3
......................................................................aagcaccagaguauacAagggc....................  43    1  Fm3
......................................................................aCgcaccagaguauaccagggc....................  2     1  Fm3
......................................................................aagAaccagaguauaccagggc....................  1     1  Fm3
```

Star Mature

auaccuggaaucaagaugugcauccuaucuaguguccuguuauauuauggcugcuauuggaaacuugaccaagcaccagaguauaccagggcagcacugggacacccacagc			
....aUgcaccagaguauaccagggc....................	2	1	Fm3
....aagcaccagaguauaccaCggc....................	1	1	Fm3
....aagcaNcagaguauaccagggc....................	1	1	Fm3
....aagcaccagaguauacGagggc....................	13	1	Fm3
....aagcGccagaguauaccagggc....................	1	1	Fm3
....aagcaccagaguauaccagggAa...................	20	1	Fm3
....aagcaccagaguauaccagggca...................	35	0	Fm3
....aagcaccaUaguauaccagggca...................	8	1	Fm3
....aagcaccagaguauaccagggcU...................	32	1	Fm3
....aagcaccagaguauaccagggcaU..................	6	1	Fm3
....aagcaccagaguauaccagggcaA..................	1	1	Fm3
....agcaccagagCauaccagggc....................	1	1	Fm3
....aAcaccagaguauaccagggc....................	1	1	Fm3
....agcaccagaguauaccagggc....................	52	0	Fm3
....accagaguauaccagggc....................	2	0	Fm3
